# Novel Computational Technologies for Next-Generation Sequencing Data Analysis and Their Applications

**DOI:** 10.1155/2015/254685

**Published:** 2015-10-20

**Authors:** Chuan Yi Tang, Che-Lun Hung, Huiru Zheng, Chun-Yuan Lin, Hai Jiang

**Affiliations:** ^1^Department of Computer Science and Information Engineering, Providence University, 200 Sec. 7, Taiwan Boulevard, Shalu District, Taichung City 43301, Taiwan; ^2^Department of Computer Science and Communication Engineering, Providence University, 200 Sec. 7, Taiwan Boulevard, Shalu District, Taichung City 43301, Taiwan; ^3^School of Computing and Mathematics, Computer Science Research Institute, University of Ulster, Jordanstown Campus, Shore Road, Newtownabbey, County Antrim BT37 0QB, UK; ^4^Department of Computer Science and Information Engineering, Chang Gung University, No. 259, Wenhua 1st Road, Guishan District, Taoyuan City 33302, Taiwan; ^5^Department of Computer Science, Arkansas State University, 2105 Aggie Road, Jonesboro, AR 72401, USA

## 1. Introduction

Next-generation sequencing (NGS) technologies, such as Illumina/Solexa, ABI/SOLiD, and Roche/454 Pyrosequencing, are revolutionizing the acquisition of genomic data at relatively low cost. NGS technologies are rapidly changing the approach to complex genomic studies, opening a way to the development of personalized drugs and personalized medicine. NGS technologies use massive throughput sequencing to obtain relatively short reads. NGS technologies will generate enormous datasets, in which even small genomic projects may generate terabytes of data. Therefore, new computational methods are needed to analyze a wide range of genetic information and to assist data interpretation and downstream applications, including high-throughput polymorphism detections, comparative genomics, prediction of gene function and protein structure, transcriptome analysis, mutation detection and confirmation, genome mapping, and drug design. The creation of large-scale datasets now poses a great computational challenge. It will be imperative to improve software pipelines, so that we can analyze genome data more efficiently.

Until now, many new computational methods have been proposed to cope with the big biological data, especially NGS sequence data. Also, many successful bioinformatics applications with NGS data through these methods have unveiled a lot of scientific results, which encourage biologists to adopt novel computing technologies. The research papers selected for this special issue represent recent progress in the aspects, including theoretical studies, novel algorithms, high performance computing technologies, and method and algorithm improvement. All of these papers not only provide novel ideas and state-of-the-art technologies in the field but also stimulate future research for next-generation sequencing data analysis and their applications.

## 2. Computational Genomics

Development of efficient algorithms for processing short nucleotide sequences has played a key role in enabling the uptake of DNA sequencing technologies in life sciences. In particular, reassembly of human genomes (*de novo* or reference guided) from short DNA sequence reads has had a substantial impact on health research.* De novo* assembly of the genome of a species is essential in the absence of a reference genome sequence. The paper by I. Birol et al. entitled “Spaced Seed Data Structures for* De Novo* Assembly” introduces the data structure designs for spaced seeds in the form of paired *k*-mers to solve the limitation of the de Bruijn graph (DBG) paradigm for long reads. First, Bloom filter is used to store spaced seeds, and it can be tolerant of sequencing errors. Then they used a data structure for tracking the frequencies of observed spaced seeds.

Next-generation sequencing technologies are now producing multiple times the genome size in total reads from a single experiment. This is enough information to reconstruct at least some of the differences between the individual genome studied in the experiment and the reference genome of the species. However, in most typical protocols this information is disregarded and the reference genome is used. The paper by K. Buza et al. entitled “RECORD: Reference-Assisted Genome Assembly for Closely Related Genomes” proposes a new approach that allows researchers to reconstruct genomes very closely related to the reference genome (e.g., mutants of the same species) directly from the reads used in the experiment. Their approach applies* de novo* assembly software, called “RECORD,” to experimental reads and so called pseudoreads and uses the resulting contigs to generate a modified reference sequence. In this way, it can very quickly, and at no additional sequencing cost, generate new, modified reference sequence that is closer to the actual sequenced genome and has a full coverage.

## 3. Metagenomics

Characterizing the taxonomic diversity for the planet-size data plays an important role in the metagenomic studies, while a crucial step for doing the study is the binning process to group sequence reads from similar species or taxonomic classes. The metagenomic binning remains a challenge work because of not only the various read noises but also the tremendous data volume. The paper by Y.-C. Lin entitled “A New Binning Method for Metagenomics by One-Dimensional Cellular Automata” introduces an unsupervised binning method for NGS reads based on the one-dimensional cellular automaton (1D-CA). The proposed method facilitates reducing the memory usage because 1D-CA costs only linear space.

## 4. High Performance Computing

The Smith-Waterman (SW) algorithm has been widely utilized for searching biological sequence databases in bioinformatics. However, the SW is a time-consuming algorithm and its usage may be limited by the sequence length and the number of sequences in a database. The previous works related to SW on GPGPU cannot solve the protein database search problem for the next-generation sequencing applications well. The paper by Y. Liu et al. entitled “Accelerating Smith-Waterman Alignment for Protein Database Search Using Frequency Distance Filtration Scheme Based on CPU-GPU Collaborative System” proposes an efficient SW alignment method, called CUDA-SWfr, for the protein database search by using the intratask parallelization technique based on a CPU-GPU collaborative system. Before doing the SW computations on GPU, a procedure is applied on CPU by using the frequency distance filtration scheme (FDFS) to eliminate the unnecessary alignments.

Compound comparison is an important task for the computational chemistry. By the comparison results, potential inhibitors can be found and then used for the pharmacy experiments. The time complexity of a pairwise compound comparison is O(*n* ^2^), where *n* is the maximal length of compounds. The intrinsic time complexity of multiple compound comparison problem is O(*k* ^2^ *n* ^2^) with *k* compounds of maximal length *n*. The paper by C.-Y. Lin et al. entitled “Accelerating Multiple Compound Comparison Using LINGO-Based Load-Balancing Strategies on Multi-GPUs” proposes a GPU-based algorithm for MCC problem, called CUDA-MCC, on single- and multi-GPUs. Four LINGO-based load-balancing strategies are considered in CUDA-MCC in order to accelerate the computation speed among thread blocks on GPUs.

## 5. Genomics

The Circular Pattern Matching (CPM) problem appears as an interesting problem in many biological contexts. CPM consists in finding all occurrences of the rotations of a pattern *P* of length *m* in a text *T* of length *n*. The paper by Md. A. R. Azim et al. entitled “SimpLiFiCPM: A Simple and Lightweight Filter-Based Algorithm for Circular Pattern Matching” presents SimpLiFiCPM, a simple and lightweight filter-based algorithm to solve CPM problem. Much of the speed of the proposed algorithm comes from the fact that our filters are effective but extremely simple and lightweight.

Rapid advances in high-throughput sequencing techniques have created interesting computational challenges in bioinformatics. One of them refers to management of massive amounts of data generated by automatic sequencers. Therefore, an efficient data model to deal with a very large amount of nonconventional data, especially for writing and retrieving operations, is very important. The paper by R. Aniceto et al. entitled “Evaluating the Cassandra NoSQL Database Approach for Genomic Data Persistency” discusses the Cassandra NoSQL database approach for storing genomic data. They perform an analysis of persistency and I/O operations with real data, using the Cassandra database system.

## 6. Conclusion

All of the above papers address either novel computational strategies or applications for next-generation sequencing data. They also develop related method and approach improvements in applications of computational genomics and database. Honorably, this special issue serves as a landmark source for education, information, and reference to professors, researchers, and graduate students interested in updating their knowledge about or active in next-generation sequencing data analysis, metagenomics, computational genomics, and database system.

